# Spontaneous Loculated Bilateral Hydropneumothoraces in a Patient with Recent COVID-19 Infection

**DOI:** 10.1155/2022/3284857

**Published:** 2022-05-26

**Authors:** Jacques Lowe, Bradley Kaptur, Ali Baltaji, Daniel Rosenblat, James Kumar, Vishesh Paul

**Affiliations:** ^1^Carle Illinois College of Medicine, University of Illinois Urbana-Champaign, Champaign, IL, USA; ^2^Department of Internal Medicine, Carle Foundation Hospital, Urbana, IL, USA; ^3^Department of Pulmonology, Carle Foundation Hospital, Urbana, IL, USA

## Abstract

A 53-year-old male presented to the emergency room with chest pain, shortness of breath, and back pain. He had recently recovered from COVID-19 infection and returned home on room air. Chest imaging showed bilateral hydropneumothoraces that were not present on the imaging performed during his prior admission three weeks ago. The patient was treated with bilateral chest tube drainage and oxygen support and responded well to treatment. This case represents a unique occurrence of spontaneous loculated bilateral hydropneumothoraces in the context of recent clinical recovery from COVID-19 infection requiring inpatient treatment. This case highlights the importance of an awareness of a potential sequela of COVID-19 that may occur even after presumed clinical recovery.

## 1. Introduction

Despite being nearly two years into the COVID-19 pandemic, we continue to see novel disease presentations and complications. While common symptoms include fatigue and dyspnea, more severe complications may include pulmonary, vascular, and neurological manifestations [1]. Of additional interest are late complications of COVID-19 that present with new symptoms. Currently, there are isolated case reports of sequelae occurring late in the disease state or even after clinical resolution and inpatient discharge.

The incidence of pneumothorax, in particular, has been of interest to researchers, and previous works have estimated that this complication may occur in 1% of patients with COVID-19 requiring hospital admission [2]. It is more commonly seen as a manifestation of barotrauma in patients on mechanical ventilation; however, some reports have discussed increased incidence even in nonintubated patients with ARDS [3, 4]. Loculated pneumothorax refers to a pneumothorax with localized air trapping within the pleural space [5]. They occur typically due to pleural pathology, including empyema, parapneumonic effusions, malignancy, or incomplete pleurodesis [6]. A hydropneumothorax is defined by the presence of both air and fluid in the pleural space. Bilateral presentation of pneumothorax is less common than unilateral presentation, with a reported incidence of approximately 1.6% of pneumothoraces [7]. Most pneumothoraces are attributable to some known causative factor, with spontaneous pneumothorax being rarer and traditionally associated with tall and thin male patients [8].

Here, we present a case of delayed presentation of bilateral spontaneous hydropneumothoraces in a patient who had recently recovered from COVID-19 infection. This is the first case of a unique presentation of this complication to the best of our knowledge.

## 2. Case Presentation

A 53-year-old male presented to an outside hospital with worsening shortness of breath, chest pain, and back pain. He had been hospitalized for 6 days at another hospital, three weeks ago, due to COVID-19 infection. At that time, his symptoms included headaches, fever, nausea, and shortness of breath. During this hospital stay, his COVID-19 symptoms ranged from mild to moderate severity. He required neither ICU admission nor mechanical ventilation, and there were no significant complications during the hospital stay. He had received remdesivir and dexamethasone, and as the symptoms improved, he was discharged home on room air. Initially, he continued to improve for a week after discharge. However, he slowly began to experience progressive chest pain, back pain, and productive cough, which prompted him to return to the emergency department.

His past medical history was relatively uneventful. He denied smoking and drug use, although he endorsed occasional alcohol use. He denied any ongoing health issues or previous surgeries. He had infrequently sought medical attention in the past.

**Figure 1:**
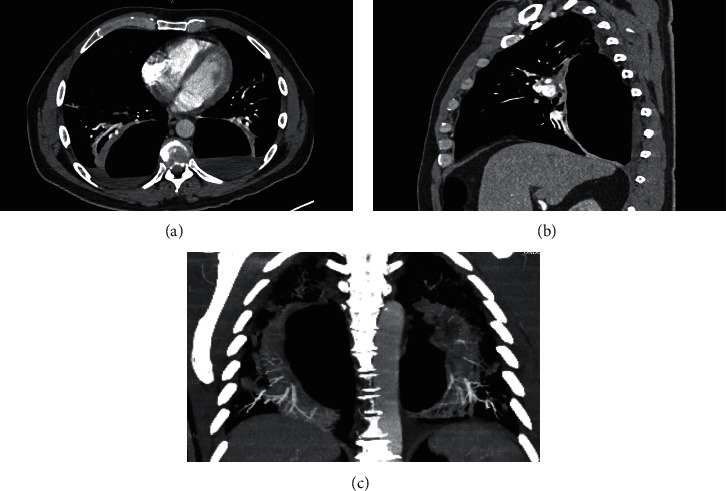
CTA from the outside hospital imaged prior to transfer demonstrating large cystic lesions in the (a) axial, (b) sagittal, and (c) coronal views.

**Figure 2:**
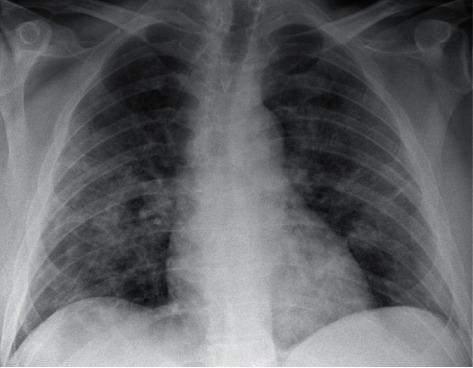
Anteroposterior chest radiographs of the patient during COVID-19 infection approximately 1 month prior to the current hospital admission.

Upon arrival at the outside hospital, he was febrile and hypoxic and required supplemental oxygen to maintain SpO_2_ >90%. CT scan performed was negative for pulmonary embolism but revealed bilateral ground-glass infiltrates consistent with recent COVID-19 pneumonia. The imaging also showed bilateral posterior lung lesions with air-fluid levels, suspicious for lung abscesses (Figure 1). Compared to his prior imaging from a few weeks ago, these lesions were new (Figure 2). At this point, the patient was transferred to our institution for further pulmonary and infectious disease workup. Upon presentation to our emergency room, the patient continued to endorse pleuritic chest pain and back pain. His vital signs were as follows: a temperature of 36.7°C, blood pressure of 147/78, heart rate of 92, respiratory rate of 22, and SpO_2_ of 93% on room air. He could speak in complete sentences and required 3 L/min supplemental oxygen to maintain normal SpO_2_. His physical exam was significant for decreased lung sounds posteriorly on auscultation bilaterally.

The patient was admitted to the medical unit for diagnostic workup and medical management. The complete blood count (CBC) showed a white blood cell count of 10,400/mm^3^ with 70% neutrophils, a hemoglobin level of 15.3 g/dL, and platelets of 352 × 10^3^/*μ*L. Serum chemistries, liver function tests, and procalcitonin levels were within normal limits.

The initial differentials included infectious etiologies that cause lung abscesses, including bacterial, fungal, and mycobacterial infections. An extensive infectious disease workup including respiratory cultures, fungal serologies, and HIV and acid-fast smears was conducted, and results were grossly negative. Bronchoscopy and bronchoscopic alveolar lavage were also negative for identification of infectious etiology. Upon further review of chest films with our radiologist, it was determined that the air and fluid levels were in the pleural space and the patient had bilateral loculated hydropneumothoraces. On further questioning, the patient denied any recent trauma and surgical interventions.

Once the diagnosis of bilateral loculated hydropneumothoraces was made, traumatic and iatrogenic causes were considered. However, the patient denied any history of trauma and recent lung-related procedures. A congenital problem was also unlikely, as chest imaging did not have these lesions a few weeks prior.

**Figure 3:**
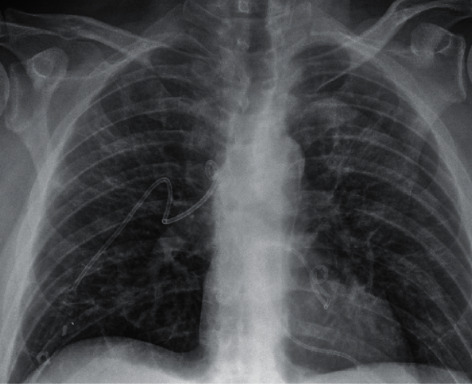
Anteroposterior chest radiograph confirming chest tube placement.

Broad-spectrum antibiotics were started initially, but they were discontinued as the infectious workup returned negative. Once the diagnosis of loculated hydropneumothoraces was confirmed, bilateral 8.5-French pigtail catheters were placed into the patient's posterior left and right hemithoraces under ultrasound guidance (Figure 3). Analysis of the drained pleural fluid was performed. No malignant cells were identified; reactive appearing mesothelial cells and blood were noted. The fluid consisted of fluid protein 4.9 g/dL, albumin 2.9 g/dL, amylase 17 U/L, cholesterol 130 mg/dL, lactate dehydrogenase (LDH) 285 U/L, and triglycerides 47 mg/dL. A pathogen panel on the pleural fluid was grossly negative, and cultures of the fluid demonstrated no growth.

A continuous air leak was present in both the chest tubes for the first 3-4 days, raising the concern for bronchopleural fistulas. The thoracic surgery team contemplated surgical intervention, but the air leak began to slow down after the fourth day. Despite the improvements in the air leak, however, the chest tube could not be removed because the cavities continued to reappear whenever suction was discontinued. Repeat chest imaging, two weeks later, showed that the pneumothoraces had decreased in size but were not entirely resolved. Eventually, the patient was discharged home on room air with bilateral chest tubes attached to Heimlich valves.

**Figure 4:**
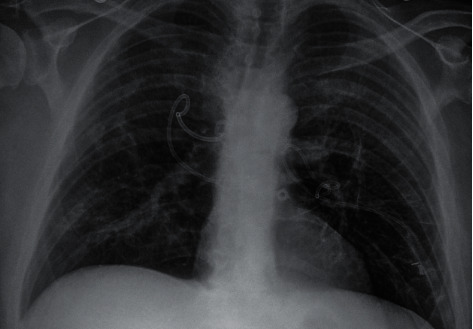
Anteroposterior chest radiograph taken 1 week after chest tube placement.

**Figure 5:**
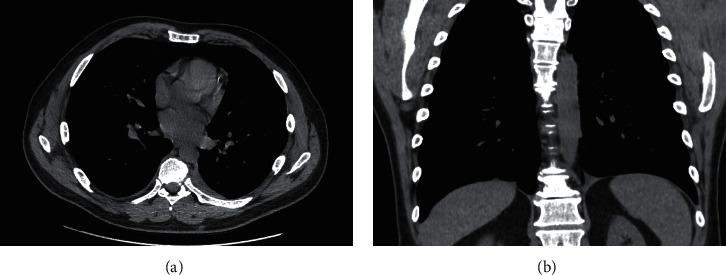
Repeat CT demonstrating resolution of hydropneumothoraces in the (a) axial and (b) coronal views.

Repeat imaging was performed at regular intervals using either chest X-ray or CT (Figure 4). Repeat CT at 1 month demonstrated interval resolution of the pleural collections with only a minimal amount of the pleural fluid remaining on the left, and the chest tubes were removed at that time. Repeat CT at 6 weeks (2 weeks after tube removal) was similar to that of 4 weeks, and the hydropneumothoraces were considered resolved (Figure 5). Furthermore, routine imaging was discontinued at that time.

## 3. Discussion

Pneumothorax is a relatively rare complication of COVID-19 infection [2]. However, many reports found a higher incidence of pneumothorax and pneumomediastinum in COVID-19 compared to other causes of ARDS and proposed a few possible mechanisms [3, 4]. The significant air hunger in patients with ARDS due to COVID-19 infection leads to increased tidal volume and respiratory rates [3, 4]. Increased respiratory drive with intense patient inspiratory efforts results in large tidal volumes, which can result in major swings in transpulmonary pressures. These transpulmonary pressure changes increase the risk of air leaking outside the alveolar space (pneumomediastinum/pneumothorax) [9]. Large tidal volume swings and changes in transpulmonary pressures also perpetuate a form of lung injury called patient self-inflicted lung injury (P-SILI) [10].

While the possible mechanisms of pneumothorax in the context of an ongoing COVID-19 infection have been described, there has been substantially less discussion of the mechanisms and risk factors of pneumothorax after clinical recovery from COVID-19. Review of the recent literature demonstrates that there have been only a handful of cases of a spontaneous pneumothorax after clinical recovery from COVID-19. Notably, some of these cases have occurred in a patient without any underlying risk factors for pneumothorax [11,12]. Thus, this complication appears to have the potential to develop in the absence of traditionally implicated risk factors, which include the presence of active pulmonary disease, treatment using mechanical ventilation, or smoking status [13]. Similarly, there are also insufficient data currently to determine whether vaccination status is protective of this particular late complication in patients who do develop COVID-19.

Our case presents a unique variation of this complication that is noteworthy for two reasons. First is the delayed presentation, i.e., the patient presented after clinical resolution of the acute COVID-19 infection. Second is the atypical radiological characteristics, i.e., it is rare for a patient to have bilateral loculated spontaneous hydropneumothoraces in any context, and ours may be the only reported case of such a complication after COVID-19 infection.

We could not confidently ascertain the etiology of delayed pneumothoraces even upon detailed history and exam. The patient's demographics did not fit those typical of spontaneous pneumothorax [8]. He did not have any pleural or pulmonary procedures or any form of trauma that could have led air to enter the pleural space. In addition, the pneumothoraces were not present on chest imaging performed during the previous hospitalization a few weeks prior (Figure 2) and this delayed manifestation makes this pathology even rarer. The initial radiologic read of the bilateral lung lesions was noncommittal and led to the workup of infectious etiologies. However, reviewing the imaging again with the chest radiologist established that the abnormalities were in the pleural space.

While there is no conclusive way to say that bilateral loculated spontaneous hydropneumothoraces were due to the patient's recent COVID-19 infection, the temporal relationship of the illness and the abnormalities a few weeks later, the absence of any other apparent causes, and other reports of increased risk of pneumothorax in COVID-19 patients justify this attribution as highly likely.

The medical community is still just beginning to understand the possible short- and long-term sequelae of COVID-19 infections. As clinicians continue to study the widespread effects of the virus and its lingering impact, it is plausible and unfortunate that such seemingly rare complications may become more commonplace.

## 4. Conclusion

Documentation in the literature of the spectrum of possible complications of COVID-19 is vital, as we are still learning more about the varied presentations of COVID-19. Researchers have advocated for the importance of both monitoring and counseling in the context of recovery from COVID-19 [1]. This case, among others reporting on sequelae of known COVID-19 infections, provides evidence to justify resource allocation toward continued outpatient monitoring and primary care visits in the weeks and months following resolution of an inpatient stay for COVID-19.

## Data Availability

No data were used to support this study.
